# Modeling and Selection of RF Thermal Plasma Hot-Wall Torch for Large-Scale Production of Nanopowders

**DOI:** 10.3390/ma12132141

**Published:** 2019-07-03

**Authors:** Liuyang Bai, Jiaping He, Yuge Ouyang, Wenfu Liu, Huichao Liu, Haizi Yao, Zengshuai Li, Jun Song, Yinling Wang, Fangli Yuan

**Affiliations:** 1 Zhumadian Academy of Industry Innovation and Development, Huanghuai University, Zhumadian 463000, China; 2 State Key Laboratory of Multi-phase Complex Systems, Institute of Process Engineering, Chinese Academy of Sciences, Beijing 100190, China; 3 Jiangsu Jingyuan Environmental Protection Co., Ltd, Nantong 226001, China

**Keywords:** numerical simulation, thermal plasma, hot-wall torch, nanopowder synthesis

## Abstract

Fouling is a great problem that significantly affects the continuous operation for large-scale radio-frequency (RF) thermal plasma synthesizing nanopowders. In order to eliminate or weaken the phenomenon, numerical simulations based on FLUENT software were founded to investigate the effect of operation parameters, including feeding style of central gas and sheath gas, on plasma torches. It is shown that the tangential feeding style of central gas brings serious negative axial velocity regions, which always forces the synthesized nanopowders to “back-mix”, and further leads to the fouling of the quartz tube. Moreover, it is shown that sheath gas should be tangentially fed into the plasma reactor to further eliminate the gas stream’s back-mixing. However, when this feeding style is applied, although the negative axial velocity region is decreased, the plasma gas and kinetic energy of the vapor phase near the wall of the plasma reactor are less and lower, respectively; as a result, that plasma flame is more difficult to be arced. A new plasma arcing method by way of feeding gun instead of torch wall was proposed and put in use. The fouling problem has been well solved and plasma arcing is well ensured, and as a result, the experiment on large-scale production of nanopowders can be carried out for 8 h without any interruption, and synthesized Si and Al_2_O_3_ nanopowders exhibit good dispersion and sphericity.

## 1. Introduction

With high enthalpy, high temperature, good heat, and electric conductivity, RF thermal plasma provides a unique environment for chemical reaction and nanopowder synthesis distinct from the ordinary solid, liquid, and gas phases, which makes it a versatile technique that is capable of controlling the morphology, size, and chemical composition of the nanopowders through the design of experimental devices and their operating parameters [1,2,3,4,5,6]. The plasma torch has the most important role of the plasma device where the plasma flame is generated and the nanopowders are synthesized. The structure of the torch has great effects on the generation and stability of the plasma flame and further influences its application in nanopowder synthesis. A plasma torch consists of three gas streams in the actual operations, including central gas (working as plasma gas), sheath gas (protecting the quartz tube), and carrier gas (carrying raw powders). Different inlet styles in the feeding of central gas and sheath gas are applied such as axial feeding [7,8,9,10] and tangential feeding [11,12,13] to get more appropriate plasma flame in the application of RF thermal plasma techniques. During the past decades, thermal plasma synthesis of nanopowders was thoroughly studied and the nanopowders synthesized by plasma showed excellent properties and were attractive for applications in different fields [14,15,16]. Therefore, improving the plasma technique and equipment for large-scale production of nanopowders has become an urgent problem to be solved at present. Fouling inside the plasma torch is one of the main obstructions of continuous plasma production of nanopowders.

Compared with traditional methods, numerical simulation can reduce the costs of the experimental cycle and provide lots of information that cannot be dynamically observed by experimental work, such as velocity fields, concentration distributions, physical properties, and so forth. A direct numerical simulation of the plasma torch makes it visualized, clarifies the fluid fields inside the plasma torch, and captures these impacts, which probably brings exciting results [17,18]. Therefore, numerical simulation has been widely used in momentum transfer [19,20], heat transfer [21,22], and reactor design [23,24,25]. Colombo et al. [26,27] founded three-dimensional models to investigate particles’ motion and sheath gas stream’s mixing inside the plasma reactor. Ye et al. [28] built models to characterize the behavior of chemically reactive species in a nonequilibrium inductively coupled argon–hydrogen thermal plasma under pulse-modulated operation. Xue et al. [29] applied a two-dimensional electromagnetic model based on FLUENT code to study the fluid and temperature fields in an inductively coupled plasma reactor.

In the present work, numerical models are developed to simulate the RF thermal plasma torch with different inlet styles of central gas and sheath gas. By comparing the calculation results, the effects of gas streams’ inlet styles on the fluid fields inside the plasma reactor can be obtained, which helps find the best inlet style of gas streams to avoid the fouling of the plasma torch. Selection of gas inlet styles and design of the plasma torch are guided by the numerical simulation, and the associated problems are also examined and solved.

## 2. Numerical Model of Plasma Torch

### 2.1. RF Thermal Plasma Reactor

**Figure 1:**
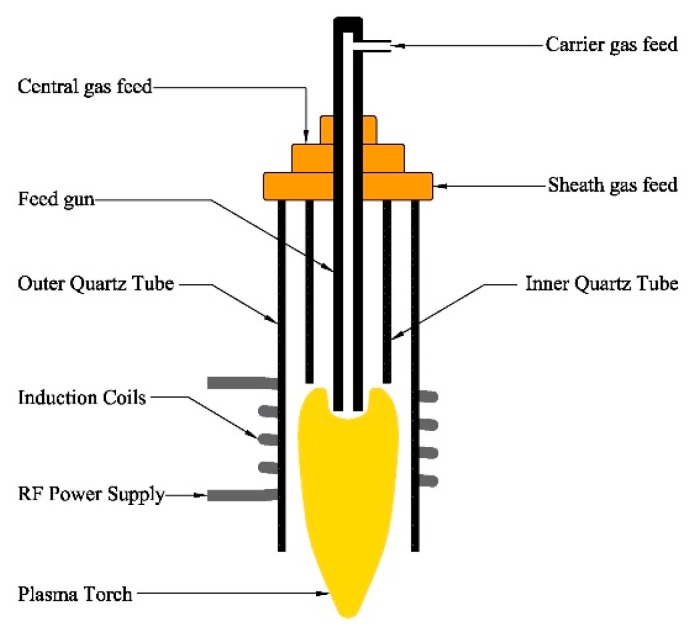
RF thermal plasma reactor.

Figure 1 is the present RF thermal plasma reactor, consisting of induction coils, inner quartz tube, outer quartz tube, feed gun, and necessary support structures. Other equipment, such as RF power, cooling chamber, collector, vacuum system, and so forth, are not involved in Figure 1. In the plasma synthesizing nanopowders, central gas and sheath gas are indispensable to provide plasma gas and protect the quartz tube from being burned, and carrier gas carries raw materials into the plasma torch.

For continuous production of nanopowders, there are many problems that need to be solved, such as sphericity morphology, and especially the reactor’s fouling. When fouling appears, the feed gun is easily blocked, the quartz tube is easily burned, and there is a greater possibility for discharge phenomena to occur. Among them, the feed gun’s fouling, resulting from the gas streams’ backflow, is representative, because it can quickly block the feed gun in a few seconds, greatly reducing the continuous operation cycle. Therefore, it is necessary for flow fields inside the plasma reactor, affected by the flow patterns of central gas and sheath gas, to be investigated.

### 2.2. Physical Models

**Figure 2:**
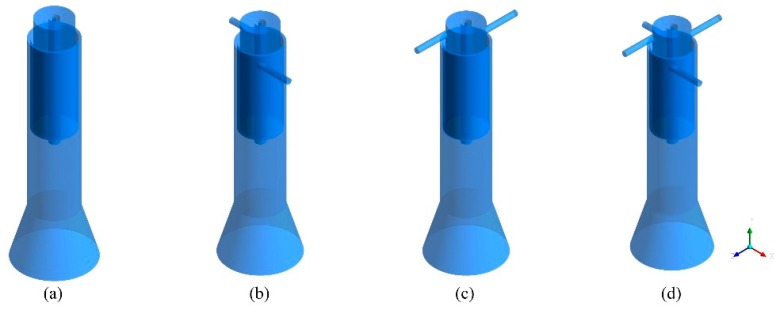
Physical models of RF thermal plasma torch: (**a**) Case A, (**b**) Case B, (**c**) Case C, (**d**) Case D.

In this work, three-dimensional models are applied to simulate the RF thermal plasma torch based on the actual equipment in our laboratory, which can fully consider axial velocity, radial velocity, and tangential velocity. Many research works have proven that the inlet styles of central gas and sheath gas can be axial feeding (gas stream flows without tangential motion) and tangential feeding (gas stream flows with tangential motion). To consider all inlet styles, four physical models are applied in numerical calculations, which are fully axial feeding (Case A), axial feeding of the central gas (Case B), axial feeding of the sheath gas (Case C), and fully tangential feeding (Case D). In addition, as the models are focused on the feed gun’s fouling problem, the calculation domains are defined as quartz tube area and reaction area. Therefore, the bottom calculation boundaries are all defined as the bottom surface of the reaction area. Based on these conditions, the physical models can be obtained, which are listed in Figure 2.

### 2.3. Calculation Specifications

In the actual experimental works, the plasma reactor is operated at atmospheric conditions, thus the pressure is set as 0. The temperature of each inlet gas stream is defined as room temperature, 20 °C. Moreover, with precalculation of the Reynolds number of each gas stream, it is found that the flows are all in turbulent zone. Since there are some streams with tangential inlet style in these models, there is strong circumfluence in the plasma torch. Therefore, turbulence models in FLUENT are set as k-ε standard model to consider turbulence effect and circumfluence effect.

### 2.4. Calculation Assumptions

As the RF thermal plasma torch and reactor are complex equipment, the calculations are simplified and several assumptions are listed below:

(1) Axial inlet style assumes that the gas stream is uniformly fed into the annular cross-section of the top of quartz tube, while tangential inlet style assumes that the gas stream is uniformly fed into the round cross-section of the branch tube.
(2) Environmental temperature is assumed as a constant value, 20 °C. It means that the effects of environment on temperature are ignored and that environmental temperature cannot be affected by the plasma reactor.
(3) Radiation in the plasma reactor is defined as the DRTM radiation model, which can be easy to converge and can improve calculation accuracy by increasing the number of rays.

## 3. Simulations for Fouling Elimination

### 3.1. Boundary Specifications

To investigate fluid fields of different inlet styles with plasma torches, sheath gas and carrier gas are fed into the plasma reactor. In this part, the operating parameters, including components, physical properties, and so forth, except for the flow rates of sheath gas and carrier gas, are also kept the same. The specific flow rates of each model are listed in Table 1.

### 3.2. Temperature Fields and Velocity Fields

**Figure 3:**
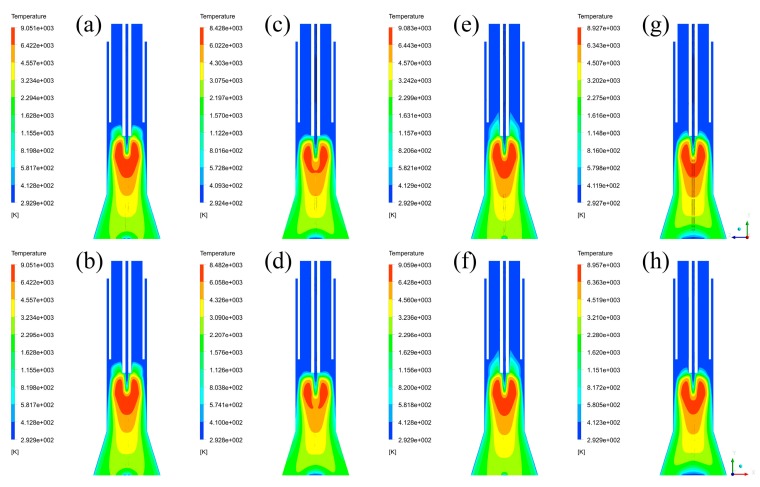
Temperature fields: (**a**) Case A in YZ plane, (**b**) Case A in XZ plane, (**c**) Case B in YZ plane, (**d**) Case B in XZ plane, (**e**) Case C in YZ plane, (**f**) Case C in XZ plane, (**g**) Case D in YZ plane, (**h**) Case D in XZ plane.

With the convergence of iterative calculations, temperature fields with XY plane and YZ plane can be captured, which are listed in Figure 3. By comparing the temperature fields of each case in different planes, it is found that the distributions of temperature are the same, and that their differences are the maximum temperature of the plasma reactor. The differences of the maximum temperature in different planes are ranging from 0 to 54 K, occupying a very small part in the global temperature range, thus it can be neglected. It means that temperature is a uniform parameter in different cross-sections of the plasma reactor. By comparing the maximum temperature between different cases (Case A: 9051 K, Case B: 8428 K, Case C: 9083 K, Case D: 8927K), it is shown that the models with axial feeding of the central gas have lower maximum temperature, while the models with axial feeding of the sheath gas have higher maximum temperature. These differences mean that tangential feeding of the central gas and axial feeding of the sheath gas can improve the energy density of plasma torches. However, the maximum temperature difference of the maximum temperature in the four cases is 655 K, possessing a small proportion in the global range of temperature, about 7.7%. This percentage means that the inlet styles can only affect the temperature distributions in a small range.

**Figure 4:**
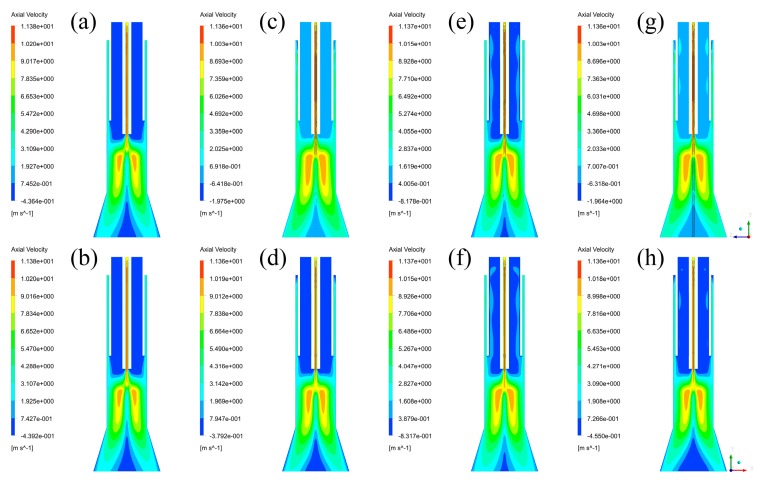
Axial velocity fields: (**a**) Case A in YZ plane, (**b**) Case A in XZ plane, (**c**) Case B in YZ plane, (**d**) Case B in XZ plane, (**e**) Case C in YZ plane, (**f**) Case C in XZ plane, (**g**) Case D in YZ plane, (**h**) Case D in XZ plane.

Nanopowders’ motion is greatly affected by gas streams, and thus it is necessary to obtain velocity fields of every case inside the plasma reactor. As the total velocity fields in three dimensions cannot provide intuitive data in a two-dimensional image, axial velocity fields, which are firstly captured and listed in Figure 4, and radial velocity fields are applied to replace the total velocity field. By comparing the axial velocity fields in different planes, it is found that their positive velocity regions are the same, while their negative velocity regions are obviously different. As the reactor models of Case B–D are not uniform in the XZ plane, the position of branch tubes can significantly affect the velocity fields near the outlet nozzle of these branch tubes. Furthermore, the velocity regions of Case B ranging from −0.3792 m/s to −1.975 m/s, and Case D ranging from −0.4550 m/s to −1.964 m/s, are very small, which means that they can be neglected. Therefore, it is indicated that the axial velocity fields of each case in different planes can be regarded as the same, and that different cross-sections have no significant effect on the distributions of axial velocity.

Moreover, by comparing axial velocity fields in Figure 4, it is shown that maximum axial velocity of the four cases are each 11.38 m/s, 11.36 m/s, 11.37 m/s, 11.36 m/s, and that the minimum axial velocity are each −0.4392 m/s, −0.3792 m/s, −0.8317 m/s, −0.4550 m/s, despite the effect of branch tubes. Since maximum and minimum axial velocity can each represent positive axial velocity distributions and negative axial velocity distributions, this result is separated into two parts to be discussed. For one, the difference of maximum axial velocity of each case is less than 0.02 m/s, which is a very small part in comparison with the maximum axial velocity. In addition, by comparing the positive axial velocity regions of each case, it can be found that the positive axial velocity regions are nearly distributed in the same area. It means that there is no significant effect of inlet styles on the positive axial velocity in the plasma reactor. For another, the maximum difference of minimum axial velocity in each case is 0.4525 m/s, which is already bigger than the absolute value of the minimum axial velocity in Case B. It is indicated that the inlet styles can affect negative axial velocity distributions in a large range. Since negative axial velocity regions are mainly located inside the quartz tube, their effects need to be discussed in detail, and are shown in the next part. In general, it can be concluded that the positive axial velocity fields of different cases are nearly the same, and that the differences of the axial velocity fields between different inlet styles are focused on the negative axial velocity regions.

**Figure 5:**
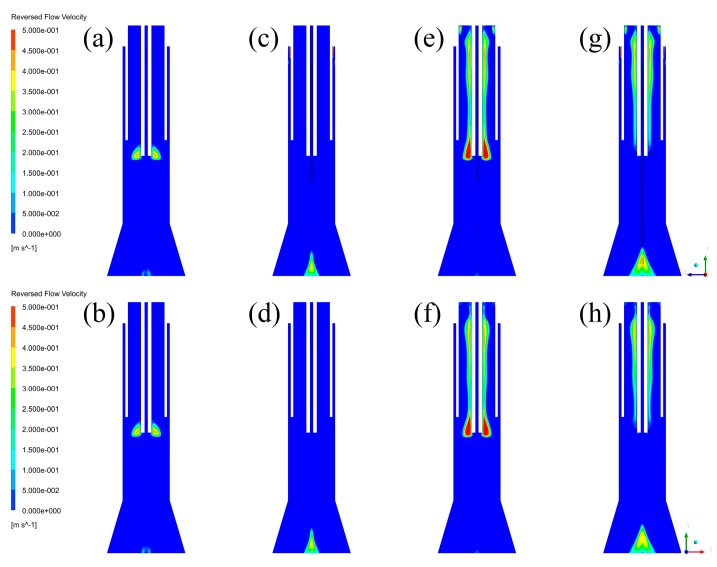
Reversed flow velocity fields: (**a**) Case A in YZ plane, (**b**) Case A in XZ plane, (**c**) Case B in YZ plane, (**d**) Case B in XZ plane, (**e**) Case C in YZ plane, (**f**) Case C in XZ plane, (**g**) Case D in YZ plane, (**h**) Case D in XZ plane.

To make clear the effects of inlet styles on negative axial velocity fields in the plasma reactor, the reversed flow velocity fields, ranging from 0 to 0.5 m/s, are captured and listed in Figure 5. As the fields of each plane are the same, the influence of different cross-sections on the fields is no longer discussed here. By comparing the reversed flow fields of each case, it can be found that the reversed flow regions are mainly distributed inside the inner quartz tube, and that only a small part of reversed flow regions are located at the bottom of the models. Since the bottom of the models are also the calculation boundaries of each model, the reversed flow regions near this area are mainly affected by the calculation boundaries. Thus, the existence of these regions can be ignored and the main investigated objects are the regions inside the inner quartz tube.

By comparing Case C to Case D (or Case A to Case B) in Figure 5, it is found that tangential feeding of the sheath gas can reduce the reversed flow regions. In addition, by comparing Case B to Case D (or Case A to Case C) in Figure 5, it is found that axial feeding of the central gas can also reduce the reversed flow regions. Thus, it is concluded that tangential feeding of the sheath gas and axial feeding of the central gas are beneficial to reduce the reversed flow regions, and vice versa. Furthermore, since the reversed flow regions are very close to the powder injector, if any particles flow into these reversed flow regions, the particles can be forced to “back-mix”, causing the fouling phenomenon of the inner quartz tube. In general, the effects of inlet styles on negative axial velocity fields are very important, and can help to reduce the possibility of fouling the inner quartz tube in the case of injecting particles.

**Figure 6:**
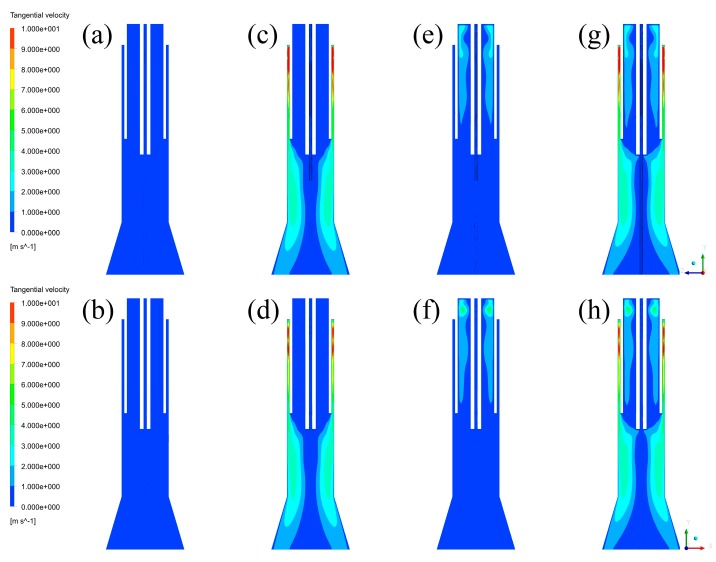
Tangential velocity fields: (**a**) Case A in YZ plane, (**b**) Case A in XZ plane, (**c**) Case B in YZ plane, (**d**) Case B in XZ plane, (**e**) Case C in YZ plane, (**f**) Case C in XZ plane, (**g**) Case D in YZ plane, (**h**) Case D in XZ plane.

The effects of inlet styles on negative axial velocity fields can be explained by the existence of tangential velocity distributions, which are listed in Figure 6, and the expansion of gas streams. It is seen from Figure 6 that there are strong tangential velocity regions in Case B–D, while Case A has no tangential velocity region because of no tangential gas stream. Since central gas with different inlet styles and sheath gas with different inlet styles have different effects, their effects are each discussed below. For central gas, the existence of tangential velocity can form strong circumfluence of gas streams, which can create strong centrifugal forces to the vapor phase. It can urge central gas toward the wall of inner quartz, which can then form a relative vacuum region near the wall of the powder injector. Since the bottom of the inner quartz tube is the boundary between low temperature and high temperature, the gas streams can be strongly expanded by the heat of the plasma torches. After these two processes, the expansion of gas streams can force the vapor phase to flow into the vacuum region, forming the negative axial velocity fields. For sheath gas, the existence of tangential velocity can force sheath gas to move towards the wall of the quartz tube, which leaves a bigger space for central gas and carrier gas. Thus, the expanded gas streams have larger space to flow past, which means that the negative velocity regions can be reduced by sheath gas with tangential inlet style. Since the existence of tangential velocity cannot significantly decrease the pathway of sheath gas, the effect is slight, which can be seen from Figure 5. With the analysis of central gas and sheath gas, it can be concluded that the negative velocity fields can be significantly increased by the tangential velocity of central gas, and slightly reduced by the tangential velocity of sheath gas.

**Figure 7:**
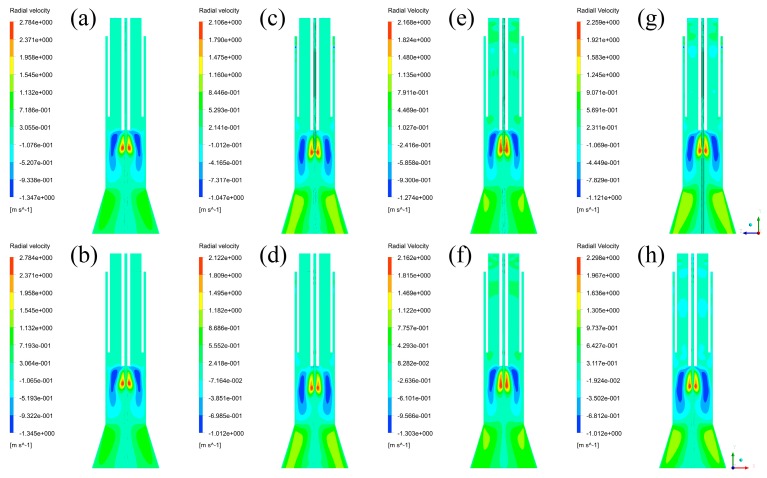
Radial velocity fields: (**a**) Case A in YZ plane, (**b**) Case A in XZ plane, (**c**) Case B in YZ plane, (**d**) Case B in XZ plane, (**e**) Case C in YZ plane, (**f**) Case C in XZ plane, (**g**) Case D in YZ plane, (**h**) Case D in XZ plane.

To obtain further information about the effects on velocity fields in the plasma reactor, radial velocity fields are then captured and listed in Figure 7. By comparing radial velocity fields of different planes in the four cases, it is shown that the distributions are nearly the same. The velocity regions are distributed in the same area, and the differences of their maximum radial velocity are ranging from 0 to 0.039 m/s, which can be neglected. By comparing the radial velocity fields between the four cases, it can be found that their maximum radial velocities are each 2.784 m/s, 2.105 m/s, 2.168 m/s, 2.259 m/s, which means that Case A has the strongest radial velocity field and Case B has the weakest radial velocity field. Since the high radial velocity regions are very small, it means that inlet styles can affect the radial velocity distributions in a small range.

### 3.3. Discussion of Fluid Fields and Their Effects

Since plasma technology is often used in powder treating, the parameters inside the plasma reactor need to be noted, including axial velocity fields, radial velocity fields, temperature fields, and so forth. These parameters can affect particles’ residence time, diffusion, and thermal process, which are all very important for powder preparations. For axial velocity field, the differences of the four cases are focused on negative velocity fields, which can force some particles to “back-mix”. The back-mixing particles will lead to the fouling phenomena of the quartz tube. Thus the existence of negative velocity fields needs to be eliminated or reduced. Since it has been concluded above that tangential feeding of the sheath gas and axial feeding of the central gas are beneficial to decrease negative velocity regions, it is better to apply the inlet styles of Case B to eliminate the effect of negative velocity regions. For radial velocity field, its existence can force the particles to diffuse towards the wall of the reactor. As the high-temperature area of the plasma reactor is distributed in the central area, the particles should be trapped in the central area to absorb sufficient energy. Thus strong radial velocity fields are obviously unfavorable for particles’ thermal process, and vice versa. As the strongest case and weakest case are Case A and Case B, respectively, it is better to apply Case B in the plasma reactor to decrease the diffusion of particles. For temperature field, the most important point is the energy density. Relatively low maximum temperature often means more uniform energy density, which is necessary for powder treating. Since axial feeding of the central gas and tangential feeding of the sheath gas can reduce the maximum temperature, the model in Case B is appropriate to be applied. What needs to be noted is that different inlet styles affect temperature distributions in such a small range that the impacts of temperature on powder treating can be neglected. With consideration of all the parameters that have been discussed above, axial feeding of the central gas and tangential feeding of the sheath gas are suggested to be applied in the process of powder treating.

A plasma torch was designed and fabricated based on the calculation selection, which is the Case B in Figure 2. However, when the torch was equipped within a plasma synthesis reactor, it was difficult to arc through the quartz torch wall using an electric spark gun. Another simulation for plasma generation had to be conducted accordingly, in which only central gas was fed into the plasma reactor because the sheath gas was always given after the arcing stage in a typical experimental procedure.

## 4. Simulations for Plasma Generation

### 4.1. Boundary Specifications

In this part, sheath gas and carrier gas are set as null to investigate the fluid fields inside the plasma reactor without plasma arc. As there are four cases that need to be calculated, some parameters need to be set as the same, including the components (pure argon), properties (constant value), pressure (0 gauge), and so forth. Their differences are the volume flow rate of each inlet boundary, accompanied by the mass flow rate, which are listed in Table 2.

### 4.2. Velocity Fields and Turbulence Kinetic Energy Distributions

**Figure 8:**
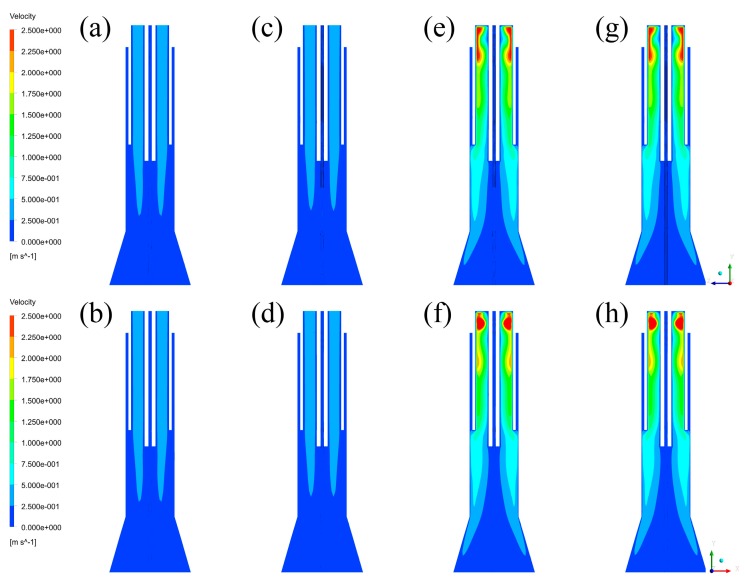
Velocity fields without plasma torch: (**a**) Case A in YZ plane, (**b**) Case A in XZ plane, (**c**) Case B in YZ plane, (**d**) Case B in XZ plane, (**e**) Case C in YZ plane, (**f**) Case C in XZ plane, (**g**) Case D in YZ plane, (**h**) Case D in XZ plane.

After the calculations of each case, velocity fields are firstly captured to analyze the differences of velocity distributions, which are shown in Figure 8. By comparing the velocity fields of YZ plane and XY plane in each case, it is indicated that there is no difference between different cross-sections of the plasma reactor. It means that central gas is uniformly filling the inner area of the quartz tube in three dimensions. However, by comparing the velocity fields between different cases, it is easily found that the velocity of Case A and B is obviously lower than that of Case C and D. In addition, it can also be found that high velocity regions of Case A and B are mainly located at the central area, while the regions of Case C and D are near the wall of the reactor. Since the inlet styles of central gas are each axial (Case A, B) and tangential (Case C, D), it is indicated that the main flow paths of central gas in the inlet styles of axial feeding and tangential feeding are each the central area and the wall region.

**Figure 9:**
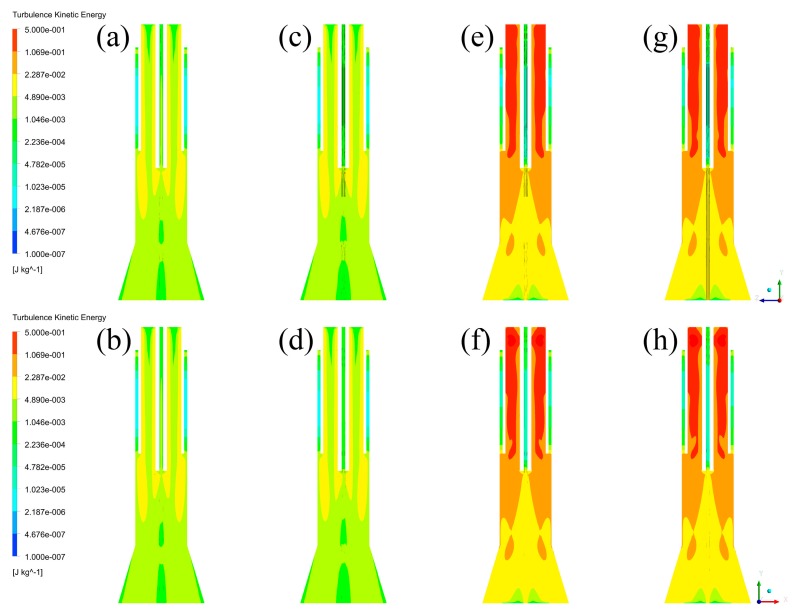
Turbulence kinetic energy distributions: (**a**) Case A in YZ plane, (**b**) Case A in XZ plane, (**c**) Case B in YZ plane, (**d**) Case B in XZ plane, (**e**) Case C in YZ plane, (**f**) Case C in XZ plane, (**g**) Case D in YZ plane, (**h**) Case D in XZ plane.

Moreover, since the turbulence kinetic energy can also represent the status of the vapor phase, energy distributions are then captured and listed in Figure 9. Similar to the velocity fields, the energy distributions cannot be affected by different cross-sections of the plasma reactor. By comparing the energy distributions between each case, it is illustrated that the turbulence kinetic energy of the vapor phase near the wall of the plasma reactor in Case C and D (>0.02287 J/kg) is obviously higher than that in Case A and B (>0.00489 J/kg). Furthermore, the high-level kinetic energy regions in Case C and D are distributed near the wall, while the regions in Case A and B are located at the central area. Therefore, it is concluded that the models with tangential feeding of the central gas have higher kinetic energy near the wall of the plasma reactor.

### 4.3. Discussions of Fluid Fields and Their Effects

Plasma arcing is a very important question for plasma technology. In the actual operation of plasma arcing, an electric spark generator is firstly placed near the bottom of the induction coils to provide sparks to the plasma gas through the quartz wall. Since RF power can come from a strong magnetic field inside the quartz tube, the plasma torch can be arced by the spark. To make it easier to be arced, continuous feeding of plasma gas and enough kinetic energy are necessary. The former—plasma gas—can be considered as the fuel of the plasma reactor, and the latter—kinetic energy—can be regarded as combustion-supporting. Since the electric spark is placed outside the quartz tube, the distribution and kinetic energy of plasma gas near the wall of the quartz tube can reflect the difficulty of plasma arcing more directly. As the velocity fields and kinetic energy distributions have been discussed above, they can help to compare the difficulty in the process of plasma arcing.

Based on the calculation results, it can be seen that tangential feeding can form higher velocity and higher kinetic energy near the wall of the plasma reactor in comparison with axial feeding. On one hand, as the gas streams are mainly passing through the high-velocity regions in the plasma reactor, it can be regarded that tangential feeding can force the gas to distribute in the wall region of the plasma reactor, which is in contrast with axial feeding. It means that central gas with tangential feeding has more plasma gas for arcing. On the other hand, since the energy distributions of tangential feeding near the wall of the plasma reactor are obviously higher than those of axial feeding, it is illustrated that tangential feeding has more kinetic energy for plasma arcing. Therefore, it is concluded that the plasma torch can be more easily arced in the conditions of tangential feeding of the central gas than in those of axial feeding of the central gas.

## 5. Experimental Verification of Simulation Results

According to the simulation above, Case B in Figure 2 is the best choice in continuous steps that can well weaken the fouling problem, and Case C and Case D in Figure 2 are the best choices in the plasma arced step. However, the fouling problem is not well solved in Case C and Case D, and the problem of torch fouling after a long time of operation inhibits us from using the easy-arcing torch. Therefore, a new plasma arcing method by way of feeding gun instead of torch wall was proposed and put in use, in order to adapt to the new torch structure, which was designed for axial feeding of central gas and tangential feeding of sheath gas. In the plasma arcing step, the electric spark gun comes into contact with the top of the feeding gun and discharges. The electric spark enters the coil induction area through the feeding gun and lights the plasma arc. With application of the new arcing method, the fouling problem is well solved and plasma arcing is ensured.

In a typical experimental procedure for Si nanoparticle synthesis, the reactor system was first purged with Ar to get rid of oxygen and water, because a separate nonoxidative atmosphere was desired during the experimental process. Plasma flame was generated using argon as both plasma-forming gas and sheath. Si nanoparticles were synthesized using Si micropowders as raw materials via a physical vapor deposition process with no chemical reactions involved, in which Si micropowders were evaporated in the high-temperature plasma flame and cooled down afterwards. However, a small amount of hydrogen was used as carrier gas to guarantee the nonoxidative environment and the high purity of the products. In a typical experimental procedure for Si nanoparticle synthesis, plasma flame was generated using argon as plasma-forming gas and oxygen as sheath. Al_2_O_3_ nanoparticles were synthesized using metallic Al micropowders as raw materials via a chemical vapor deposition process, in which Al was oxidized by oxygen to form Al_2_O_3_. Oxygen was applied as both sheath gas and carrier gas.

Typical parameters are given in Table 3. Si micropowders were provided by Hebei Tianjing Photovoltaic Technology Co., Ltd (Hengshui, China). Al micropowders were provided by Hebei Jisheng Aluminum Powder Co., Ltd (Xinji, China). Argon and oxygen were provided by Beijing Qianxi Jingcheng Gas Sales Center (Beijing, China).

The reactor was heated by the plasma flame for about 5 min until the system reached a steady level, and then the raw materials were injected into the plasma flame in a continuous way. The raw materials were supplied by a homemade screw feeder and fed axially through an injection probe to the top of the plasma flame by carrier gas, and the products were collected by a high-temperature filter collector with automatic cleaning function. In order to protect the products from oxidizing at high temperature when Si nanoparticles were synthesized, extra Ar supply was necessary until the reactor system was cooled down.

**Figure 10:**
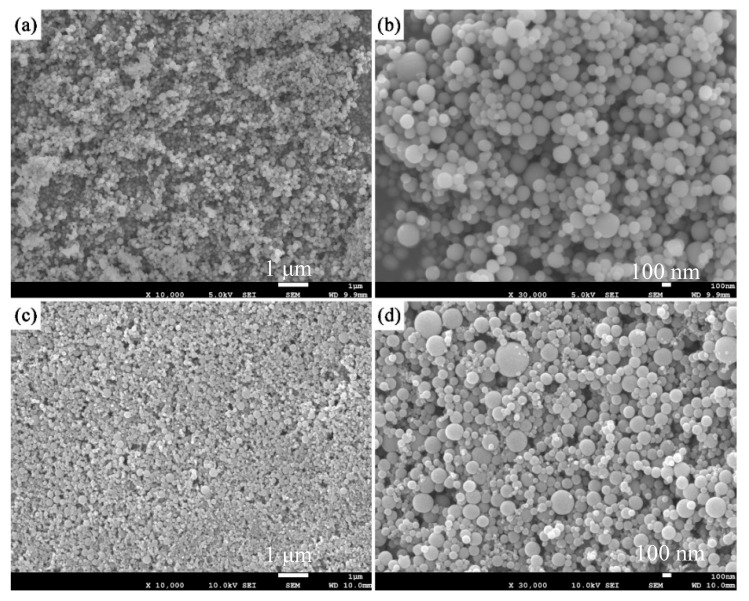
FESEM images of the large-scale produced nanopowders: (**a**,**b**) Si, (**c**,**d**) Al_2_O_3_.

The collected products were inspected with field emission scanning electron microscopy (FESEM, JEOL JSM-6700F, JEOL, Akishima, Japan). Figure 10 presents FESEM images of the large-scale produced nanopowders. Si nanopowders were synthesized via a physical vapor deposition in Ar–H_2_ (volume ratio of 15/1) plasma, and Al_2_O_3_ nanopowders were synthesized by way of oxidation of aluminum powders in Ar-O_2_ (volume ratio of 3/5) plasma. Figure 10a,b are FESEM images of synthesized Si nanoparticles with different magnification, and Figure 10c,d are FESEM images of synthesized Al_2_O_3_ nanoparticles with different magnification. One can notice that both Si and Al_2_O_3_ nanopowders consisted of uniform particles with good dispersion and sphericity. The particle size was about 100 nm, and there existed more coarse particles in Al_2_O_3_ samples than in Si samples. The exothermic reaction within the synthesis of Al_2_O_3_ would have caused the particle growth and the existence of coarse particles. Fouling had been significantly weakened and continuous operation cycle of large-scale production of nanopowders was extended from 2–3 h (continuous operation cycle of unoptimized synthesizing processes of nanopowders) to as long as 8 h.

## 6. Conclusions

In order to solve the fouling problem encountered during the continuous operation for large-scale production of nanopowders, the models with different inlet styles of central gas and sheath gas are founded to investigate their effect on the fluid fields inside the plasma reactor. The following conclusions can be drawn from the discussions:

(1) The models with different inlet styles have approximate temperature fields and positive axial velocity fields, and have different negative axial velocity fields and radial velocity fields. It means that the selection of torch structure will not cause any unpredictable influences on temperature fields.
(2) Axial feeding of the central gas and tangential feeding of the sheath gas can decrease negative axial velocity regions and reduce radial velocity intensity, which can help eliminate the fouling of the quartz tube and increase the efficiency of energy utilization.
(3) The inlet style of tangential feeding can provide more plasma gas into the area of the torch inner wall and higher kinetic energy of vapor phase in comparison with axial feeding. It means that axial feeding would make it difficult to arc through the quartz torch wall using an electric spark gun.
(4) A plasma torch with axial feeding of the central gas and tangential feeding of the sheath gas was selected for large-scale production of nanopowders, and a new plasma arcing method by way of feeding gun was proposed and put in use, in order to adapt to the new torch structure.
(5) Fouling had been significantly weakened, and the continuous operation cycle of large-scale production of nanopowders was extended to 8 h. Synthesized Si and Al_2_O_3_ nanopowders exhibited good dispersion and sphericity.

## Figures and Tables

**Table 1:** Gas flow rate set in each inlet boundary.

	Vapor Phase Parameters	Carrier Gas	Central Gas	Sheath Gas
Case A	Mass flow rate/g·s^−1^	0.0924	0.4618	1.8471
	Volume flow rate/m^3^·h^−1^	0.20	1.00	4.00
Case B	Mass flow rate/g·s^−1^	0.0924	0.4618	0.9235
	Volume flow rate/m^3^·h^−1^	0.20	1.00	2.00
Case C	Mass flow rate/g·s^−1^	0.0924	0.2309	1.8471
	Volume flow rate/m^3^·h^−1^	0.20	0.50	4.00
Case D	Mass flow rate/g·s^−1^	0.0924	0.2309	0.9235
	Volume flow rate/m^3^·h^−1^	0.20	0.50	2.00

**Table 2:** Central gas set in each inlet boundary.

	Mass Flow Rate/g·s^−1^	Volume Flow Rate/m^3^·h^−1^
Case A	0.4618	1.00
Case B	0.4618	1.00
Case C	0.2309	0.50
Case D	0.2309	0.50

**Table 3:** Typical experiment parameters.

Parameters	Values
Central gas	1.00 m^3^·h^−1^
Sheath gas	2.00 m^3^·h^−1^
Carrier gas	0.20 m^3^·h^−1^
Powder feedrate	10.0 g·min^−1^
